# Alumnol

**Published:** 1893-05-20

**Authors:** 


### NEW DRUGS AND PREPARATIONS.

### ALUMNOL.

Among the newer antiseptics alumnol has been shown to possess remarkable activity, rivalling carbolic acid in its power of penetrating deeply into tissues and at the same time having valuable astringent and sedative effects.

It is a salt of aluminium with a naphthol sulpho-acid, and forms a fine white powder of acid reaction, fairly soluble in cold, and freely soluble in hot water. It has been shown by Heinz and Liebrecht to have the power of destroying the spores of anthrax and other microbes in twenty-four hours in even a one per cent. solution, while in the strength of four parts in a thousand it stops the growth of typhus, cholera, Finkler-Prior, and anthrax bacilli. The curious property which it possesses of coagulating albumen, while the precipitate redissolves at once in an excess of albumen, enables it not only to act as a surface antiseptic, but to penetrate deeply into the tissues.

For disinfecting suppurating sinuses it can be used at the strength of one per cent., or, if a slightly caustic effect is admissible, up to 20 per cent. In gonorrhœa great success has been obtained by employing injections of one per cent. three times a day for several days, and then using only one half or a quarter of that strength for a longer period. Heinz used it in three hundred cases after this method with striking success. In gonorrhœal-endometirtis pencils, containing from two to five per cent., were introduced with satisfactory results.

The effect of the drug on wounds in half and one per cent. solution is very marked. The previously existing suppuration rapidly disappears, and the wound gradually heals up. Moreover, alumnol has such extraordinary penetrative power that the deeper tissues are reached more easily than by most other antiseptics, and the skin around old ulcers can be thoroughly purified by its use.

It would also seem to be non-poisonous when imbibed at the strength usually employed for lotions. Chotzen employed it extensively in affections of the skin, both acute and chronic. It was applied either as a lotion of from 2½ to 10 per cent. dissolved in alcohol for eczema and psoriasis on exposed surfaces, or as an ointment made with Lanoline, and containing from 2 to 20 per cent.; or, again, as a protective varnish. It appeared of special value from its penetrating powers in chronic types of skin disease with great thickening, and where much infiltration has been produced below the surface. In cases of these kinds dressings containing from 10 to 20 per cent. were found most efficient. Heinz was particularly impressed by the relief afforded in several acute skin affections through the application of a protective varnish containing alumnol. It has recently been employed in affections of the throat and nose with good results by Spengler. Solutions of from a-half to 10 per cent. were applied on cotton wool daily for periods varying from a few days to some months. The 5 per cent. solution proved of great value in two cases of acute and six of chronic pharyngitis, some of them being complicated with ozæna and atrophic rhinitis. He also used it in laryngeal affections, but the results here compared badly with those by chloride of zinc, though it was less disagreeable to the patients. As an application for affections of the middle ear, it possesses the advantage of coming away in a soluble form instead of remaining as a solid mass until syringed away. In fact, there would seem to be a great future in store for this drug in aural practice alone. The comparative cheapness of alumnol is a point in which it competes successfully with similar disinfecting agents. On the whole, we may expect to find so valuable a remedy extensively employed in several departments of surgical practice.

